# Ursolic acid and rosmarinic acid ameliorate alterations in hippocampal neurogenesis and social memory induced by amyloid beta in mouse model of Alzheimer’s disease

**DOI:** 10.3389/fphar.2022.1058358

**Published:** 2022-12-22

**Authors:** Fatima Javed Mirza, Saadia Zahid

**Affiliations:** Neurobiology Research Laboratory, Department of Healthcare Biotechnology, Atta Ur Rahman School of Applied Biosciences (ASAB), National University of Sciences and Technology (NUST), Islamabad, Pakistan

**Keywords:** Alzheimer’s disease, neurodegeneration, adult hippocampal neurogenesis, rosmarinic acid, ursolic acid, DCX, Ki-67

## Abstract

Alzheimer’s disease (AD) is a multifaceted neurodegenerative disorder characterized by substantial neuronal damage which manifests in the form of deficits in memory and cognition. In spite of the debilitating nature of Alzheimer’s disease (AD), a dearth of treatment strategies calls for the need to develop therapeutic agents that stimulate neurogenesis and alleviate the associated cognitive deficits. The present study investigates the therapeutic potential of two major phytochemicals*,* rosmarinic acid (RA) and ursolic acid (UA) in an amyloid beta_1–42_ (Aβ_1–42_)-induced model of AD. UA, a natural pentacyclic triterpenoid and RA, a phenolic ester are major bioactive constituents of *Rosmarinus officinalis*, which is a medicinal herb belonging to family Lamiaceae and exhibiting significant biological properties including neuroprotection. Donepezil, a second generation cholinesterase inhibitor approved for the treatment of mild, moderate and severe Alzheimer’s disease (AD) is used as control. Out of eight groups of male BALB/c mice, stereotaxic surgery was performed on four groups (*n* = 6 each) to introduce Aβ_1–42_ in the hippocampus followed by treatment with vehicle (phosphate-buffered saline (PBS)), donepezil, UA or RA. The other four groups were given vehicle, donepezil, UA and RA only. Behavior analysis for social interaction was performed which constitutes the social affiliation and the social novelty preference test. Presence of Aβ plaques and expression of neurogenesis markers i.e., doublecortin (DCX) and Ki-67 were also assessed. Results revealed the neuroprotective effect of UA and RA observed through substantial reduction in Aβ plaques as compared to the Aβ_1-42-_ and donepezil-treated groups. The neuronal density was also restored as evident *via* DCX and Ki-67 immunoreactivity in Aβ_1–42_ + RA and Aβ_1–42_+UA-treated groups in comparison to Aβ_1–42-_treated and Aβ_1–42_+donepezil-treated groups. The social affiliation was reestablished in the Aβ_1–42_ administered groups treated with UA and RA. Molecular docking studies further validated the comparable binding of UA and RA with Ki-67 and DCX to that of donepezil. Our findings suggest that UA and RA are potential neuroprotective compounds that reverses the histological hallmarks of AD and ameliorate impaired social memory and hippocampal neurogenesis.

## Introduction

Alzheimer’s disease (AD) is the most common form of dementia accounting for more than 80% of the cases diagnosed. With a prevalence that continues to grow as the world population ages, it has emerged as a leading health problem. The debilitating disorder affects nearly 50 million people worldwide (Crous-Bou et al., 2017). It is characterized by the formation of neurofibrillary tangles (NFTs) of hyperphosphorylated tau protein and amyloid beta (Aβ) plaques which manifests as deficits in cognition and memory (Lopez et al., 2019). Aβ is a major contributing factor in neurotoxicity and neural function and the deposition of amyloid plaques in the hippocampus, cerebral cortex and amygdala can lead to stimulation of astrocytes and microglia, axonal and dendritic damage and synaptic loss which manifest as cognitive impairments (Armstrong, 2009; Chen et al., 2017). The plaque formation constitutes the primary pathological process associated with AD while NFT formation and the subsequent neurodegeneration are downstream processes (Lane et al., 2018).

Adult hippocampal neurogenesis, is a unique phenomenon hosted by the hippocampus, which confers significant levels of plasticity to the hippocampal circuitry improving pattern separation and spatial memory (Anacker et al., 2018). AD leads to a sharp decline in adult hippocampal neurogenesis in comparison to neurologically healthy subjects (Moreno-Jiménez et al., 2019). Impaired neurogenesis is thereby considered as a relevant mechanism that leads to cognitive deficit associated with AD.

Among the various markers of neurogenesis, Doublecortin (DCX) is a brain-specific protein associated with the microtubules which regulates neuronal migration through the polymerization and stabilization of microtubules in migrating neuroblasts (Sadeghi et al., 2018). DCX is vital for the proper initiation and maintenance of differentiation as well as migration during neurogenesis. Neural cells with reduced expression of DCX exhibit impaired migration, differentiation and neurite formation (Shahsavani et al., 2018). Ki-67 another widely acclaimed marker of cell proliferation and neurogenesis is expressed in dividing cells during mitosis except the G0 phase (Sun and Kaufman, 2018).

Currently, only two classes of drugs have been approved for the treatment of AD. Cholinesterase enzyme inhibitors and *N*-methyl d-aspartate (NMDA) antagonists function mainly by treating the symptoms of AD and do not possess preventive or curative effects (Breijyeh and Karaman, 2020). Although a huge amount of research on AD has been directed towards the development of disease-modifying therapy in the last decade, however there is still a dearth of therapeutic agents which will alter the course of disease rather than providing symptomatic treatment alone. Lack of disease modifying drugs even after decades of studies indicates the challenges associated with the development of therapeutic agents with curative potential against AD (Salomone et al., 2012).

In the recent times, natural compounds have garnered significant interest due to their pharmacologically significant activities. These natural products, including herbs and spices, possess various phytochemicals which serve as potential sources of natural antioxidants and neuroprotectants and are devoid of the potentially life-threatening side effects characteristic of the existing approved drugs (Twilley et al., 2020; Karthika et al., 2022). Rosmarinic acid (RA), a phenolic ester is abundantly present in the herbs belonging to the family *Labiatae* and exhibits antioxidant, antimutagenic, antiapoptotic and several other pharmacological activities (Amoah et al., 2016). It also plays a beneficial role against AD through the suppression of Aβ aggregation (Hase et al., 2019). Ursolic acid (UA), a natural pentacyclic triterpenoid also exerts health benefits against inflammation, oxidative stress and fibrosis (Wang X. T. et al., 2018; Xu et al., 2018). Also, RA and UA substantially improve the deficits in cognition as well synaptic dysregulation and the associated neurodegeneration in AD model of Aβ_1–42_ -induced neurotoxicity suggesting their therapeutic significance against AD (Mirza et al., 2021). Furthermore, an *in silico* study also presents the therapeutic potential of these compounds based on drug-likeness, pharmacokinetic properties and binding affinity with AD-associated proteins (Mirza et al., 2022).

In the current investigation we evaluated the neuroprotective effects of RA and UA against impaired neurogenesis and social memory deficits produced by Aβ_1–42_. The effects were assessed in comparison to donepezil, commonly prescribed for AD (Eskandary et al., 2019). It is routinely used as a standard drug in studies investigating the therapeutic potential of different agents against AD (Adlimoghaddam et al., 2018). Moreover computer-aided molecular docking assessment also elucidated the interaction of RA and UA with the markers of neurogenesis. The data obtained for the current study may provide further insights into molecular mechanisms and clinical intervention options for AD and its associated consequences.

## Materials and methods

### Experimental animals

Male BALB/c mice were maintained in the animal facility of Atta-ur-Rahman School of Applied Biosciences (ASAB) National University of Sciences and Technology (NUST). Mice were raised under 12 h natural light-dark cycles and had free access to food and water *ad libitum*. All animal experiments were conducted in conformity with the standards of the Institute of Laboratory Animal Research, Division on Earth and Life Sciences, National Institutes of Health, United States. The internal review board of ASAB, NUST approved the study design (IRB # 64).

### Animal model induction

BALB/c mice (male, age 10–12 weeks; 35–45 g) were used for experimentation. A previously described method was used to establish the animal model (Mirza et al., 2021). The mice were administered 50 mg/ml ketamine (80 µL) and 5 mg/ml Diazepam (60 µL) per 40 g of animal weight prior to microinjection of Aβ_1–42_ (ab120301, Abcam, Cambridge, MA, United States; dilution 1 µg per µL) into the hippocampus (CA1 region) from bregma: anteroposterior, 2.3 mm; mediolateral, 1.8 mm; dorsoventral, 2.0 mm, using a stereotaxic apparatus using a Hamilton syringe. Similarly, the control mice were administered phosphate-buffered saline (PBS). The mice were kept in individual cages until further use.

### Animal grouping

The male BALB/c mice were segregated into eight groups (*n* = 6 each). The groups 1–4 were pre-treated with Aβ_1–42_. The groups 2, 3 & 4 received donepezil (15 mg/kg) (Ahmed et al., 2017), RA (16 mg/kg; ab141450, Abcam, United Kingdom) (Farr et al., 2016), or UA (40 mg/kg; ab141113, Abcam, United Kingdom) (Liang et al., 2016), respectively for 15 days post Aβ_1–42_ administration. Group 5 received vehicle (PBS) while groups 6, 7, 8 received the similar dose of donepezil, RA and UA, respectively for similar duration. All the groups were orally administered with normal feed and water. The purity of UA and RA was >99% and >95% respectively while donepezil was taken as a positive control. After the end of treatment period the mice were subjected to behavioral tests and subsequently brain tissue harvesting.

### Social interaction behavior

The procedure was performed as reported previously (Rizwan et al., 2016). The apparatus comprised of a glass box having dimensions 40 × 40 × 40 cm with two similar cages placed inside the box. The mice placed in the cages were characterized as mouse A and B while the treated mice were referred to as test subject. Mice A and B were never encountered before with the test subject and were of the same background in terms of age, weight and gender.

#### Session I: Social affiliation test

After habituating the test subjects (5 min) inside the box, they were introduced back into the box with a cage having mouse A on one side and an empty cage on the other side. The test subject was then left in the box for 10 min and allowed to move in both the cages. The discrimination index (DI) or the interaction time or with the empty cage and mouse A was evaluated and the difference between the time spent interacting with the mouse A or empty cage and the total time was scored.

#### Session II: Social novelty preference test

The mouse B was released in the empty cage while the mouse A remained unaltered. The test subject was again allowed an exploration time of 10 min. DI or interaction time was measured as the difference between the time spent interacting with mouse A or mouse B and the total time spent with mouse B.

### Histology and immunohistochemistry

Histology was performed according to the protocol used by Amber et al., 2020. The tissue was dewaxed and rehydrated in ethanol and double distilled water (ddH_2_O). Congo red stain (1%) was applied to the tissue for 25 min after which it was thoroughly rinsed with ddH_2_O, followed by counter Haematoxylin and eosin staining. The sections were rinsed again with ddH_2_O, dried and incubated for 20–25 min before clearing in xylene solution.

Immunostaining was performed in accordance with the protocol previously described by Mirza et al., 2021. Hippocampal tissue sections (5 μm) were deparaffinized after antigen retrieval. H_2_O_2_ (1%) was applied to quench the peroxidase activity and blocked with BSA (5%) in PBS for 1 h. After an overnight incubation with primary antibodies at 4°C the sections were incubated for 1 h with a secondary antibody followed by washing thrice with TBST and visualised with 0.025% 3, 3′ diaminobenzidine (DAB Kit, ab50185, Abcam, MA, United States). Ki-67 (Leica Biosystems, PA0230) and DCX (Abcam, ab207175) diluted in the block solution 1:200 and 1:100 respectively, were used as primary antibodies while anti-rabbit IgG-HRP conjugated (ab97051, Abcam, MA, United States) diluted in block solution 1:100 was used as secondary antibody. The images were visualized using B-150, OPTIKA microscope (Italy) at 4× and 10× resolution. The images were captured using Optika Vision Lite 2.1 image analysis software. Quantitative analysis was performed for cell count in an area of 10,000 μm^2^ from three randomly selected areas and the average values were calculated and plotted.

### Molecular docking

PatchDock server was used for docking using cluster RMSD at default value of 4.0 to identify the interaction among RA and UA with target proteins DCX and Ki-67. The interaction was compared with donepezil. Patchdock algorithm produces potential complexes based on the criteria of shape complementarity (Schneidman-Duhovny et al., 2005). The 3D structures of the target proteins Ki-67 and DCX were acquired from RCSB Protein data bank (PDB) (https://www.rcsb.org/). The PDB IDs of Ki-67 and DCX were 5J28 and 2BQQ, respectively. The 3D structures of RA, UA and donepezil were taken from PubChem database (https://pubchem.ncbi.nlm.nih.gov/). The automated docking models generated were further assessed using FireDock (Mashiach et al., 2008) and visualized through BIOVIA Discovery Studio (Systèmes, 2016).

### Statistical analysis

Data are presented as mean ± SEM and statistical significance was set at 95% confidence level and a *p*-value <.05. Behavioural and biochemical data was analysed by one-way analysis of variance (ANOVA) with Bonferroni’s multiple comparisons as *post hoc test* using GraphPad Prism 5.0.

## Results

### Rosmarinic and ursolic acid improve social affiliation and social novelty preference in Aβ_1–42_ treated mice

**FIGURE 1:**
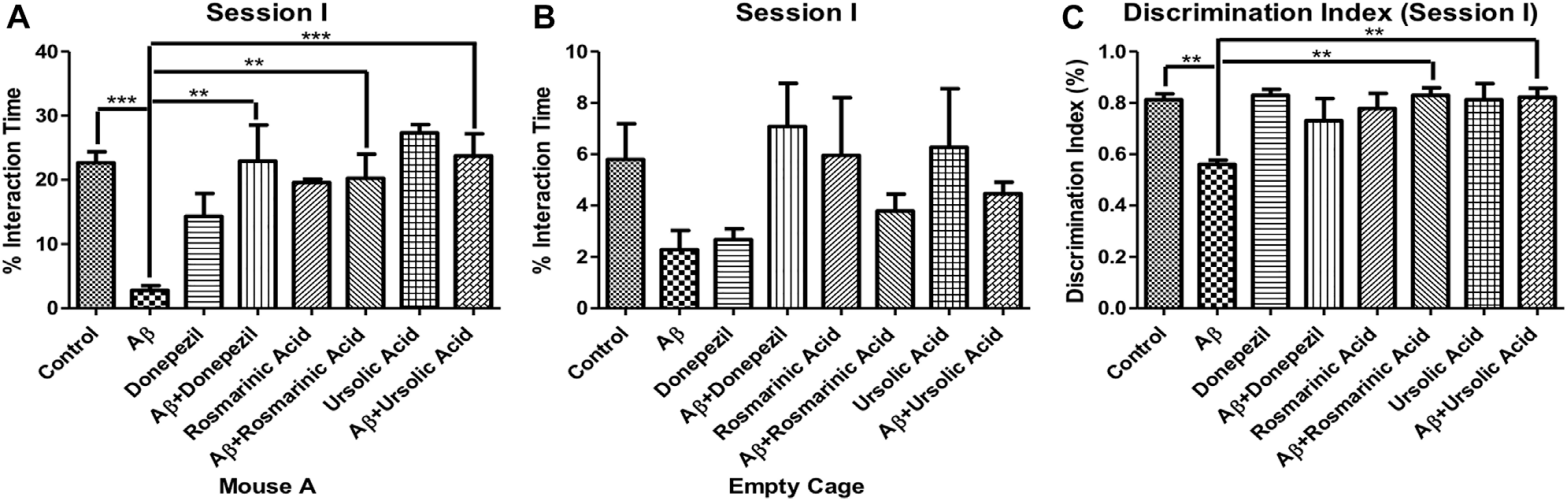
Effect of RA and UA on Social Affiliation behavior. **(A)** Interaction time with the Mouse A. **(B)** Interaction time with the empty cage. **(C)** Discrimination index during session (I). Significance was analyzed by one-way ANOVA followed by Bonferroni comparison test (mean ± SEM) using Graphpad Prism. *p < 0.05, **p < 0.01, ***p < 0.001.

To evaluate the effects of RA and on sociability in mice, social interaction test was performed. Aβ_1–42_ treated group (2.798 ± 0.7145; *p* < 0.0001) exhibited a significant reduction in interaction with mouse A, a conspecific placed in one of the cages, in comparison to the control (22.66 ± 1.726; *p* < 0.0001) and other experimental groups exhibiting a significant decrease in social affiliation. The Aβ_1–42_ + RA (20.25 ± 3.779; *p* < 0.001) and Aβ_1–42_+ UA-treated groups (23.74 ± 3.434; *p* = 0.0003) performed significantly better than the diseased group and comparable to the Aβ_1–42_+donepezil-treated group (22.91 ± 5.629; *p* = 0.0051). Aβ_1–42_+ UA-treated group exhibited improved social affiliation in comparison to all of the other groups (Figure 1).

Interactions with the empty cage also show least amount of interaction by the Aβ_1–42_ -treated group. The decrease social affiliation pattern observed during session I for Aβ_1–42_ -treated group is inferred from the same length of time spent with the mouse A (2.798 ± 0.7145; *p* < 0.0001) as well as empty cage (2.276 ± 0.7505; *p* = .0664). Conversely, the control and experimental groups interacted more with the mouse A in comparison to the empty cage (Figure 1).

The Aβ_1–42_-treated group (0.5600 ± 0.01703; *p* < 0.0001) exhibited significantly low DI in comparison to the control (0.8117 ± 0.02400; *p* < 0.0001) and the treated groups. A significant improvement in DI was observed in Aβ_1–42_ + RA (0.8300 ± 0.02887; *p* < 0.0001) and Aβ_1–42+_ UA-treated (0.8220 ± 0.03541; *p* = .0002) groups depicting restoration of social affiliation (Figure 1).

In the session II, the mice were assessed for novelty preference and social memory. The mice treated with Aβ_1–42_ showed diminished levels (6.572 ± 2.116; *p* = .0144) of interaction with an unfamiliar mouse B in comparison to the control group (21.38 ± 4.261). The treated groups did not show preference with any mouse cage.

**FIGURE 2:**
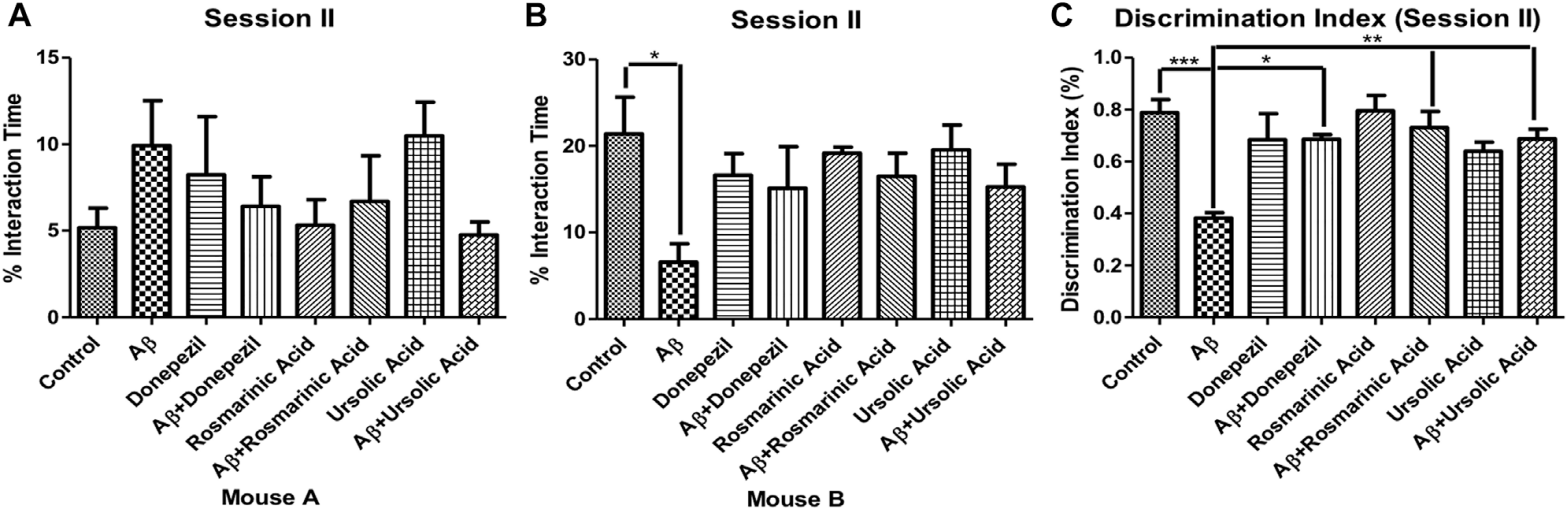
Effect of RA and UA on social novelty preference behavior. **(A)** Interaction time with the Mouse A. **(B)** Interaction time with the Mouse B. **(C)** Discrimination index during session II. Significance was analyzed by one-way ANOVA followed by Bonferroni comparison test (mean ± SEM) using Graphpad Prism. *p < 0.05, **p < 0.01, ***p < 0.001.

The DI for session II revealed a significantly low value for the Aβ_1–42_ -treated group (0.3820 ± 0.02107; *p* < 0.0001) relative to the control (0.7880 ± 0.05054; *p* < 0.0001) and the other experimental groups, thus indicating alterations in social memory. A significant improvement in DI was evident in Aβ_1–42_ + RA (0.7300 ± 0.06258 *p* = .0007) and Aβ_1–42_+ UA-treated (0.6867 ± 0.03783 *p* < 0.0001) groups in comparison to the control. The DI in RA- and UA-treated groups was comparable to that of the standard drug donepezil (0.6850 ± 0.01893; *p* < 0.0001) demonstrating their protective effects against social memory deficit in AD (Figure 2).

### Improvement in neurogenesis by RA and UA

**FIGURE 3:**
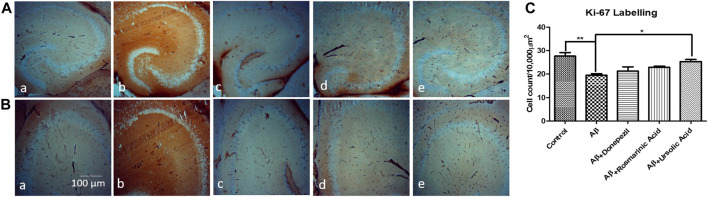
Neuron specific Ki-67 Labelling of mice hippocampal tissue: (a) Control (b) Aβ_1–42_ -treated group (c) Aβ_1–42_ + donepezil-treated group (d) Aβ_1–42_ +RA-treated group (e) Aβ_1–42_ + UA-treated groups at magnification **(A)** 4X **(B)** 10X. **(C)** Graph showing cell count/10000 µm^2^ of Ki-67 labelled cells in mice hippocampus in experimental groups respectively. Significance was analyzed by One-way ANOVA followed by Bonferroni comparison test (mean ± SEM) using Graphpad Prism. *p < 0.05 **p < 0.01.

A considerable reduction in neuronal proliferation was significantly apparent in the group treated with Aβ_1–42_ relative to the control mice. An improvement in the density of Ki-67-positive neurons was observed with treatment with RA (22.87 ± 0.4667; p= .0111) and UA (25.33 ± 0.8819; p = 0.0027) post Aβ_1–42_ administration as compared to the mice treated Aβ_1–42_ only (19.50 ± 0.6455). It was found that RA and UA have greater improvement activity than donepezil (21.30 ± 1.825; p = 0.34), evident through the restoration of the Aβ_1–42_ deteriorated neurons. No obvious neuronal loss was encountered by the control mice (27.65 ± 1.525), donepezil, RA and UA in Aβ_1–42_ untreated groups (Figure 3).

**FIGURE 4:**
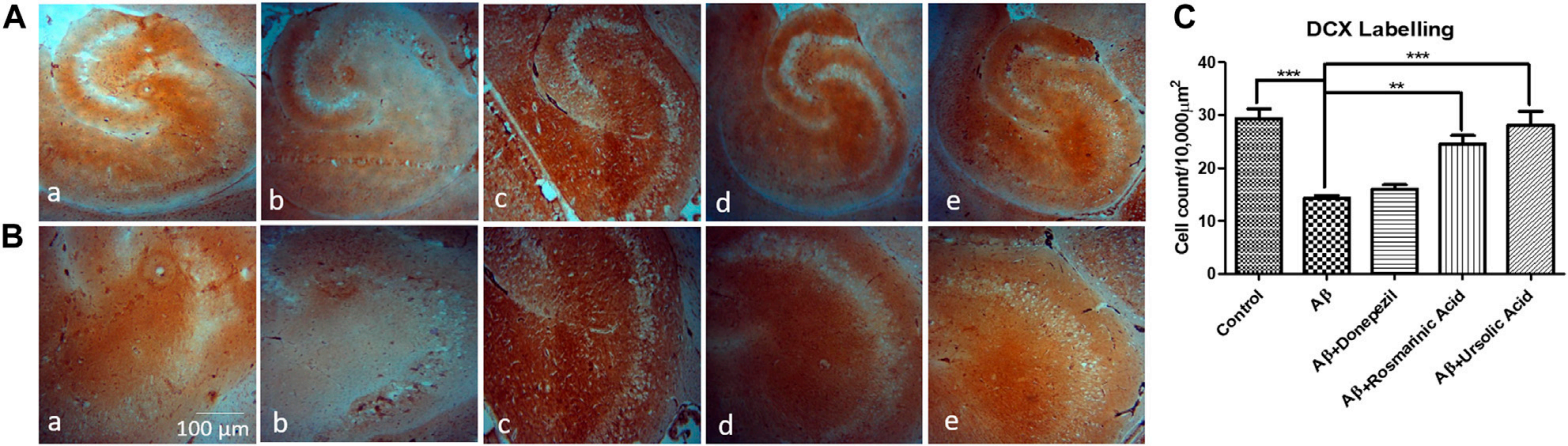
Neuron-specific DCX Labeling of mice hippocampal tissue: (a) Control (b) Aβ_1–42_ -treated group (c) Aβ_1–42_ + donepezil-treated group (d) Aβ_1-42_ + RA-treated group (e) Aβ_1–42_ +UA-treated groups at magnification **(A)** 4X **(B)** 10X **(C)** Graph showing cell count/10000 µm^2^ of DCX labelled cells in mice hippocampus in experimental groups respectively. Significance was analyzed by One-way ANOVA followed by Bonferroni comparison test (mean ± SEM) using GraphPad Prism. **p < 0.01, ***p < 0.001.

A notable decrease in the hippocampal proliferating cells of group treated with Aβ_1–42_ (14.30 ± 0.4813; p = 0.0003) relative to control (29.33 ± 1.856) indicated the intensity of neurotoxic effects of Aβ. RA (24.53 ± 1.619; p= 0.0009) and UA (28.13 ± 2.551; p= 0.0015) treatment post Aβ_1–42_ administration showed improved immunoreactivity with DCX with increase DCX positive cells relative to control and donepezil-treated group (16.07 ± 0.7881; p= .0984) (Figure 4).

### RA and UA treatment reduces the accumulated amyloid beta burden

**FIGURE 5:**
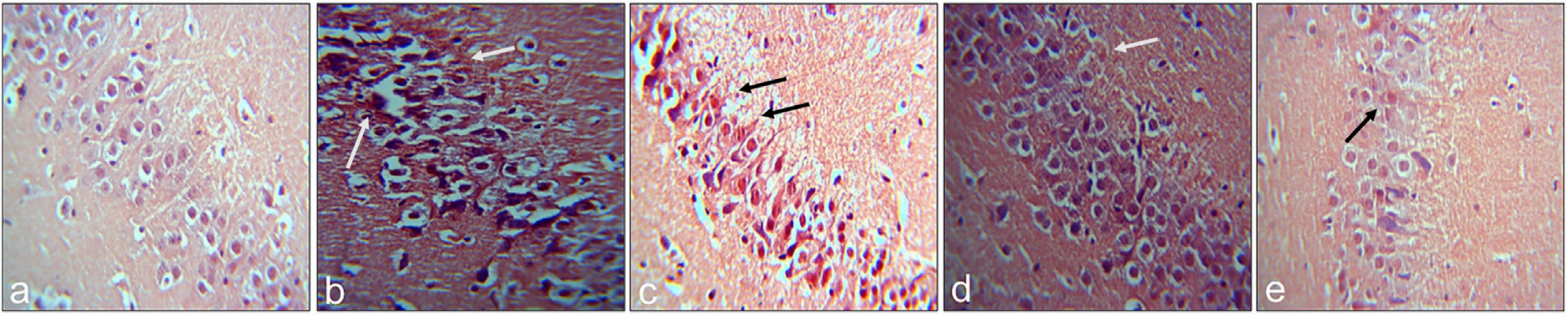
Congo red-stained sections of hippocampus for visualization of amyloid plaques: **(A)** Control **(B)** Aβ_1–42_ -treated group **(C)** Aβ_1–42_ + donepezil-treated group **(D)** Aβ_1–42_ + RA-treated group **(E)** Aβ_1–42_ + UA-treated group. Original Magnification 40X.

The presence of congophilic amyloid plaques in the mice administered with Aβ_1–42_ was substantially decreased with the treatment of RA and UA indicating a reversal of the plaque formation. RA and UA treatment significantly rescued the cellular density and morphology in Aβ_1–42_ -treated groups and comparatively have greater neuronal restoration than donepezil. The control littermates showed a normal neuronal pattern (Figure 5).

### Molecular docking studies of Ki-67 and DCX with RA, UA and donepezil

Molecular docking studies were used to predict the receptor-ligand interaction geometrics of RA, UA and donepezil with the neurogenesis markers Ki-67 and DCX. All the compounds successfully docked against the target proteins. Lowest atomic contact energy (ACE) is depictive of higher binding affinity, therefore, the ligand molecules that had the lowest ACE were considered better ligands to Ki-67 and DCX. The docking scores of the complexes are shown in Table 1. The docking results were further refined using FireDock and the results obtained are stated in Table 2.

**TABLE 1:** Protein docking scores evaluated by Patchdock.

Receptor	Ligand	Geometric shape complementarity score	Approximate interface area size of the complex (Å^2^)	Atomic contact energy (Kcal/mol)
Ki-67	Rosmarinic Acid	3652	420.90	−203.58
Ki-67	Ursolic Acid	3796	525.10	−290.71
Ki-67	Donepezil	4426	524.00	−293.37
DCX	Rosmarinic Acid	4000	467.20	−84.83
DCX	Ursolic Acid	4172	493.00	−154.20
DCX	Donepezil	4546	569.40	−157.92

**TABLE 2:** Protein docking scores refined by Firedock.

Receptor	Ligand	Global energy (kcal/mol)	Attractive vdw	Repulsive vdw	Atomic contact energy (Kcal/mol)
Ki-67	Rosmarinic Acid	−27.62	−15.86	4.81	−8.74
Ki-67	Ursolic Acid	−32.58	−14.64	7.10	−10.96
Ki-67	Donepezil	−32.99	−14.38	3.84	−10.92
DCX	Rosmarinic Acid	−25.10	−19.45	3.45	−2.38
DCX	Ursolic Acid	−31.01	−18.90	8.53	−10.06
DCX	Donepezil	−39.49	−21.83	9.16	−11.84

Vdw, Van der waal’s forces.

### UA exhibits ACE comparable to donepezil in binding interactions with Ki-67 and DCX

Binding interaction of UA, RA, and donepezil with Ki-67 revealed that amongst the other compounds, UA with an ACE of −290.71 had better affinity than RA (−203.58) which was comparable to that of donepezil (−293.37). Interactions with DCX also demonstrated a comparable binding energy of UA (−154.2) and donepezil (−157.92). These binding energy values suggest an interaction of UA with Ki-67 and DCX validating the present *in vivo* results (Table 1).

### UA shows global energy comparable to donepezil in binding interactions with Ki-67 and DCX

**FIGURE 6:**
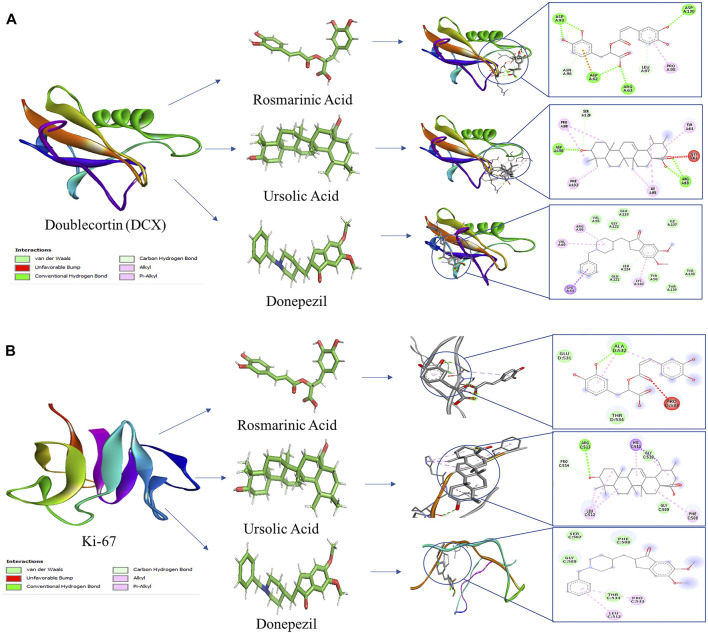
Molecular docking interaction models of RA, UA, and donepezil with DCX and Ki-67. 2D structures of the compounds are shown by line and stick models with the surrounding amino acids of **(A)** DCX and **(B)** Ki-67. The interactions are denoted by the following colors: hydrogen bonding interactions (green), alkyl bonds (pink) and bumps (red).

Global energy depicts the binding energy of the complex while the ACE is based on its contribution to global energy. Low values of ACE correspond to more stable complexes. UA exhibited an ACE of −10.96 in its interaction with Ki-67 which is comparable to that of donepezil (−10.92). UA (−10.06) also showed a comparable ACE to that of donepezil (−11.84) in its interaction with DCX. Attractive and repulsive vdw (Van der waal forces) are a quantification of the input of van der waal’s forces to the global binding energy. The values are tabulated in Table 2 and the interacting residues are shown in Figure 6.

## Discussion

Our study elucidated the potential effects of the bioactive compounds of *R. officinalis*, RA and UA on Aβ_1–42_ -induced neurotoxicity in comparison to donepezil. The data revealed the restoration capability of RA and UA of altered social memory in AD mouse models. Social engagement with the surrounding environment is associated with an improved angiogenesis, synaptogenesis, and neurogenesis which are crucial in delaying the progression of AD (Fratiglioni et al., 2004).

Notably, RA and UA are previously known to exert anxiolytic and antidepressant effects in various models of neurotoxicity (Colla et al., 2015; Ramos-Hryb et al., 2019; Lataliza et al., 2021). The present study showed that RA and UA treatment post Aβ_1–42_ administration exhibited significantly greater sociability levels relative to the diseased group. RA and UA treatment post Aβ_1–42_ administration also displayed significant improvement in social novelty preference in mice. The social interaction behavior of mice with RA and UA treatment was comparable to that of the standard drug donepezil which is indicative of their comparable protective effects to restore social memory. These data suggest the potential of RA and UA in alleviating the behavioral deficits in social affiliation and novelty preference induced by Aβ_1–42_.

Altered social behavior is demonstrated by a decrease in sociability, avoidance of novel social stimuli and exacerbation of aggression associated with amyloid pathology (Kosel et al., 2021). Impaired social interaction memory also results due to mitochondrial damage contributed to increased oxidative stress and deficits in hippocampus and medial pre-frontal cortex activity (Misrani et al., 2021). According to Okada et al., the removal of cholinergic cell groups in the basal forebrain cause an impairment of social behavior which was significantly reinstated by cholinesterase inhibitors, suggesting the critical role of cholinergic dysfunction in sociability deficits corelated with psychological and behavioral symptoms of dementia in AD (Okada et al., 2021). Interestingly, modulation of matrix metallopeptidase 9 (MMP9) caused an improvement in sociability and social recognition memory, along with a reduction in anxiety in AD mouse model, supporting the notion that targeting MMP9 could serve as a therapeutic strategy in restoring the neurobehavioral damage in AD (Ringland et al., 2021).

Although the effect of RA and UA on social memory has not been reported previously but their effect on the restoration of spatial memory and object recognition memory has been documented. A mechanistic study on RA describes its role in the inhibition of cognitive decline through the suppression of tau phosphorylation (Yamamoto et al., 2021) while UA ameliorates oxidative stress and inflammation to improve cognitive deficits in an Aβ-induced mouse model (Liang et al., 2016). UA also exerts radioprotective effects improving radiation induced deficits in memory and learning in BALB/c mice (Tang et al., 2017) whereas also exhibit a potent anti-dementia effect observed in olfactory bulbectomized mice (Nguyen et al., 2022). Improvement of spatial memory and amelioration of Aβ_25–35_ accumulation by UA has also been demonstrated (Li et al., 2020).

We further studied the effects of RA and UA on altered hippocampal neurogenesis induced by Aβ_1-42_. Several studies indicate the relevance of Aβ_1–42_ induction in the impairment of adult hippocampal neurogenesis eliciting neurodegeneration associated loss of memory and cognition. The intra-cerebrovascular injection of Aβ_1–42_ in male kunming mice caused mitochondrial damage, inflammation, loss of memory (Qi et al., 2019) and altered adult hippocampal neurogenesis observed in balb/c mice (Amber et al., 2020). In addition, the interneuronal accumulation of phosphorylated tau protein is also crucial for AD progression and impairs adult hippocampal neurogenesis through the suppression of GABAergic transmission (Zheng et al., 2020). Contrarily, promotion of neurogenesis reconstructs the degenerated neural circuits in AD hindering the associated cognitive decline (Choi et al., 2018). Aβ_1–42_ -induced neurotoxicity causes significant deterioration of Ki-67 and DCX expression levels in the hippocampal tissue (Amber et al., 2020). Our results indicated a reduction in the neuronal proliferation induced by Aβ_1–42_ evident through a significant decline in the immunoreactivity of DCX and Ki-67 positive cells in the mice treated with Aβ_1–42_, however it was considerably restored upon treatment with UA and RA.

The results of behavioral and immunohistochemical analysis indicated the neurogenic potential of UA and RA. Based on these results, *in silico* analysis was further conducted to determine the binding interactions of the compounds with the neurogenesis markers, Ki-67 and DCX in comparison to donepezil. Molecular docking analysis showed comparable binding energy values of UA to that of donepezil. UA had an ACE value comparable to that of donepezil in its interaction with Ki-67 and DCX. Previously, we reported the effect of UA and RA in normalizing the mRNA expression levels of neurogenesis markers, Ki-67, DCX and NeuN. Interestingly, UA exhibit significant restoration of the expression levels of these markers in comparison to RA and donepezil (Mirza et al., 2021). It also enhanced neurogenesis and repressed inflammation in temporal lobe epilepsy and cerebral ischemia models (Wang Y. et al., 2018; Liu et al., 2022). Its role in neurite outgrowth and neuronal survival mediated by nerve growth factor has also been reported (Theis et al., 2018). These results reiterate the neurogenic potential of UA through interaction with Ki-67 and DCX indicating its therapeutic potential against neurodegeneration associated with AD.

The reduction in the plaque formation by RA and UA post Aβ_1–42_ administration also suggests their role in the suppression of AD progression. RA has been previously found to be effective against copper (II)-induced neurotoxicity through the formation of an original ternary association between Aβ and Cu (II) (Kola et al., 2020). Also, the prevention of fibrillization and assembly of β sheets in tau protein suggest its therapeutic potential against AD (Cornejo et al., 2017). Interestingly, RA also reduces the formation of Aβ and ameliorated tissue structure in an AD-like dementia model induced by scopolamine (Deveci et al., 2021). It also exerts anti-apoptotic effect that results in the alleviation of inflammation and oxidative stress associated neurodegeneration as observed in a model for Parkinson’s disease (Lv et al., 2020). Consistently UA also hindered the deposition of Aβ and lowered the levels of its oligomers and monomers in an Aβ-induced *Caenorhabditis elegans* transgenic model (Wang et al., 2022). These results are indicative of the potential of UA and RA in the reduction of Aβ plaques which constitute a hallmark feature of AD.

## Conclusion

This study suggests the pro-neurogenic potential and neuroprotective effects of UA and RA on neurotoxicity induced by Aβ_1–42_ that represents pathological hallmarks of AD. Our findings revealed that UA and RA can rescue the AD like alterations characterized by accumulated amyloid plaques, impaired social memory and neurogenesis induced by Aβ, thereby reiterating their potential as promising therapeutic agents against AD.

## Data Availability

The original contributions presented in the study are included in the article/supplementary material, further inquiries can be directed to the corresponding author.
